# Shift Work, Jet Lag, and Female Reproduction

**DOI:** 10.1155/2010/813764

**Published:** 2010-03-08

**Authors:** Megan M. Mahoney

**Affiliations:** Veterinary Biosciences and Neuroscience Program, University of Illinois, 3639 VMBSB MC-002, 2001 S Lincoln Avenue, Urbana, IL 61802, USA

## Abstract

Circadian rhythms and “clock gene” expression are involved in successful reproductive cycles, mating, and pregnancy. Alterations or disruptions of biological rhythms, as commonly occurs in shift work, jet lag, sleep deprivation, or clock gene knock out models, are linked to significant disruptions in reproductive function. These impairments include altered hormonal secretion patterns, reduced conception rates, increased miscarriage rates and an increased risk of breast cancer. Female health may be particularly susceptible to the impact of desynchronizing work schedules as perturbed hormonal rhythms can further influence the expression patterns of clock genes. Estrogen modifies clock gene expression in the uterus, ovaries, and suprachiasmatic nucleus, the site of the primary circadian clock mechanism. Further work investigating clock genes, light exposure, ovarian hormones, and reproductive function will be critical for indentifying how these factors interact to impact health and susceptibility to disease.

## 1. Introduction

In mammals, the 24-hour clock mechanism, or circadian oscillator, is critical for the function and coordination of a broad range of biological processes, from hormone secretion to locomotor activity. This biological timing system is vital for successful reproduction. Animals are more likely to gain mating opportunities if they coordinate their sexual behavior with that of their potential partners. Females benefit from synchronizing the timing of pregnancy to seasons with favorable food and weather conditions, and it is advantageous for an animal to give birth at a time of day when it is most likely to be in a safe place such as a burrow rather than out foraging. A mounting body of evidence indicates that disruptions in normally synchronized, or entrained, biological rhythms are associated with a broad range of pathologies including reproductive dysfunction in females. 

This review will describe how the endogenous timing system interacts with the hypothalamic-pituitary-gonadal axis to regulate female reproductive cyclicity. I will address how disruptions in the alignment of these rhythms, as occurs in shift work or jet lag, are strongly associated with reproductive dysfunction in women. Animal models will highlight the relationship between circadian desynchrony and prevalence of disease states. I will lastly address how “clock genes”, the genetic components underlying the circadian mechanism, relate to reproductive function, and how hormone secretion in turn can alter clock gene rhythmicity.

## 2. Shift Work and Jet Lag

The Bureau of Labor Statistics reported that in 2004 over 27 million Americans had flexible or shift work schedules. Shift work is defined as any employment after 7 pm and before 9 am. Women working non-daytime shifts equaled 12.4% or over 3 million women. Shift work is found in services such as healthcare, military, and protection (police, firefighters). Shift-workers tend to have activity, body temperature, and hormonal rhythms that are out of phase with environmental cues and often the behavioral rhythms of their family and friends. Some workers are able to adapt or synchronize their rhythms (sleep schedules, melatonin secretion patterns) to an alternative work schedule [1]. Even so, adapting to a shift work schedule can be hindered when workers have weekends off and encounter a world operating on a standard schedule [2]. A number of individuals are never able to adjust to shift work. 

Jet lag is caused by shifts in the environmental light:dark cycle, or photic phase, that result in an organism's internal rhythms becoming transiently out of phase with the environment and each other [3]. Similar to shift work, jet lag also causes a myriad of physical, emotional, and psychiatric problems in humans [4–7]. It is likely that these disruptions in circadian rhythms are even more extreme for transmeridian travelers than those of shift workers. These individuals do not have a regular schedule to enable entrainment, they travel through different time zones which provide constantly changing light:dark schedules, and they experience light exposure at times when their internal clock mechanism indicates it should be night. All of these signals can continuously reset and disrupt internal circadian rhythms (e.g., sleep patterns). 

Shift work, jet lag, and other forms of circadian disruption including sleep deprivation increase the risk of individuals acquiring a disease or exacerbate the symptoms of a preexisting condition. Shift-work, jet lag, and sleep deprivation have been associated with an increased risk of mood disorders, depression, cardiovascular disease, endometriosis, dysmenorrhea, as well as an increased incidence and risk of breast cancer [8–11]. Shift workers and transmeridian travelers report increased fatigue and sleep disturbances relative to individuals working daytime shifts [2]. Women with chronic sleep deprivation or insomnia are more likely to have circadian rhythm disruptions and clinical depression [11]. Sleep disturbance in late pregnancy is associated with increased labor duration and increased likelihood of requiring medical intervention such as cesarean section [12]. 

These relationships between work schedules and health have gained considerable attention from society and the scientific community. The American Academy of Sleep Medicine recognizes jet lag as a sleep disorder typified by excessive daytime sleepiness and associated physiological impairments [13]. In 2007, night shift work was reclassified from a possible to a probable human carcinogen (class 2A) by the International Agency for Research on Cancer. In fact, this ruling formed the basis for a recent decision by a Danish industrial injuries board to award compensation to women shift workers. These women had worked more than 20 years as a shift worker and developed cancer [14].

## 3. The Hypothalamus-Pituitary-Gonadal Axis and the Circadian System Regulate Reproductive Cycles

**Figure 1:**
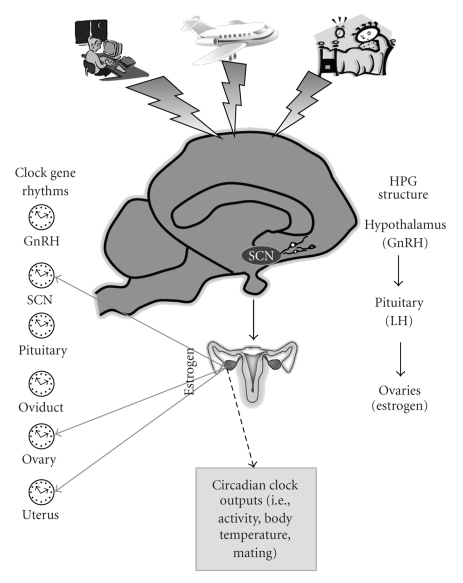
The hypothalamus-pituitary-gonadal axis regulates reproductive cycles in female mammals. Increasing levels of estrogens released from the ovaries feedback onto the hypothalamus. When estrogen stimulation reaches a threshold, gonadotropin releasing hormone (GnRH) neurons release their product into the blood stream. GnRH acts on the pituitary to trigger a surge of luteinizing hormone (LH) which then induces ovulation. In rodents, the suprachiasmatic nucleus (SCN) of the hypothalamus provides an additional signal which regulates the timing of reproductive events. Shift work schedules, jet lag, and sleep deprivation can perturb the daily (circadian) rhythms in reproduction and “clock gene” expression. Clock gene expression has been detected in the SCN, GnRH neurons and female reproductive tissues. Estrogen can influence the pattern of expression of gene expression in some of these tissues (solid arrows). Estrogen also influences the rhythmic expression of clock-controlled outputs such as activity and body temperature (dashed arrow).

Female mammals have a cyclical change in hormone secretion and ovulation. The most-studied animal models of female reproductive cyclicity are laboratory muroid rodents (i.e., rat, hamster, mouse), which have an estrous cycle characterized by a short total duration (4-5 days), spontaneous follicular development and spontaneous ovulation. The menstrual cycles of women and the estrous cycles of these rodents have several features in common including a series of tightly orchestrated events that result in increased activity in gonadotropin releasing hormone (GnRH) neurons, a hormone surge, and ovulation (Figure 1). During the follicular phase of the cycle, maturing follicles in the ovary release increasing levels of estradiol. When estradiol concentrations reach a threshold, a surge of GnRH is released from cells in the hypothalamus into the hypophyseal portal blood system. This surge of GnRH triggers a surge of luteinizing hormone (LH) from the anterior pituitary, and this hormone acts on the ovary to induce ovulation. Following ovulation the follicles rupture then are luteinized. During this cycle stage progesterone is the dominant hormone (secreted by the corpora lutea). This luteal phase lasts for 10–16 days in women. Rats and mice have corpora lutea that function for 1–3 days but do not have a true extended luteal phase without coitus or vaginal stimulation [15]. 

In rodents these reproductive events occur in a cyclical and circadian manner. In rats GnRH neurons become active just before lights-off as indicated by the presence of the immediate early gene Fos within the nucleus [16]. This rhythm is endogenous, as ovariectomized rats given steroid hormones only have a rise in GnRH cell activation at one time of day, and this rhythm persists in animals housed in constant darkness [17]. The LH surge is concurrent with the GnRH cell activation and occurs just before lights-off [18]. Ovulation occurs 6–15 hours later [19]. These latter two events occur at precise circadian intervals. For example, in hamsters housed in a light:dark cycle, the estrous cycle occurs every 96 hours (4 periods of 24 hours). When animals are housed in constant conditions this rhythm continues (range 95.35–97.54 h), indicating that an endogenous circadian mechanism (rather than the environmental light:dark cycle) is regulating the estrous cycle [20]. Rodents also exhibit a daily rhythm in the timing of mating behavior; typically this occurs around the onset of their active period [21, 22]. 

In laboratory rodents the precise timing of this cascade of estrous cycle events is regulated by a small number of cells located in a brain region called the suprachiasmatic nucleus (SCN). The SCN contains the primary circadian mechanism and regulates the timing of central and peripheral oscillators [23]. Rats and hamsters with bilateral SCN lesions lack a rhythm in sexual behavior, the preovulatory LH surge, corticosteroid rhythms, and a consistently functional estrous cycle [24–28]. Transplants of fetal SCN tissue restore some behavioral rhythms but do not restore estrous rhythms, indicating that synaptic inputs from the circadian clock are critical for mediating these systems [28].

Women also exhibit daily rhythms in the timing of reproductive cycle events and hormone secretion patterns. The preovulatory LH surge acrophase typically occurs between midnight and 8 am [29]. A second study examining the timing of the LH surge in 155 cycles (from 35 women) found that 48% of the surges occurred between 4 and 8 am and 37% of the surges occurred between midnight and 4 am [30]. In women, the precise timing of ovulation has not been determined but is estimated to occur 24–40 h later [19, 30, 31]. In humans, the LH surge occurs before the active period (daytime) as is the case for other day-active or diurnal species [32]. In nocturnal rodents, these events also occur just before their active period at the time of lights-off [21]. 

Women express other daily rhythms related to reproduction. There is diurnal variation in the pattern of pulsatile LH secretion; this rhythm of peaks and troughs remains evident even in the reduction or absence of ovarian hormones, as is seen in hypogonadal women [33]. The timing of the onset of labor and the timing of birth also exhibit strong diurnal rhythmicity with respect to time of day. The rupture of membranes is reported to occur between midnight and 4 am [34]. A study of over 17,000 term singleton deliveries found the majority of women going into labor between midnight and 8 am with 01:45 AM as the peak time of labor onset [35]. In a second study of over 15,000 women, the onset of labor had a 24-hour rhythm, with a nadir in the middle of the day and peaks around dawn and dusk [36]. The timing of birth typically occurs in the middle of the afternoon. In two retrospective studies (6608 and 15,000 women, resp.), the majority of births following a spontaneous onset of labor occurred between 1 and 2 pm [36, 37]. Interestingly, multiparous women were more likely to deliver babies earlier in the morning, between 8 and 11 am when compared to nulliparous mothers, the authors speculated this difference may be related to the timing of fetal and maternal hormone secretions [37]. A clinical study was conducted to determine if this diurnal rhythm in the onset of labor was also important to women in whom labor was induced. Women which had labor induced in the morning required less uterine stimulation (i.e., oxytocin), had a shorter interval from induction to birth, and were less likely to require operative assistance with the delivery when compared to women that had their labor induced in the evening hours [38]. 

These reproductive events in women occur with a diurnal rhythmicity; however, it remains possible that the circadian system does not control the timing of reproductive events in humans as tightly as it does in rodents. For example, humans and primates are able to copulate throughout the ovarian cycle and are not limited to a particular time of day or specific duration of exposure to steroid hormones [39]. In primates a surge in LH can be induced at any time of day with the proper strength and interval of steroid hormone treatment [40]. This does not eliminate the possibility that appropriate and successful reproduction in women is regulated by the light:dark cycle and/or a circadian timing mechanism. Nearly all of the available data indicate that alteration of the phase relationship between an animal, human or otherwise, and the light:dark cycle has adverse effects on the physiology of the affected organism. In support of this hypothesis, alterations or disruptions in the daily (and potentially circadian) rhythms of women are linked to significant disruptions in reproductive function.

## 4. Disruption of Diurnal Rhythms Is Associated with Reproductive Dysfunction

Women working an evening shift, night shift, or have irregularly scheduled shifts (such as days-off or flexible schedules) report altered menstrual cycle length (both increases and decreases), increased menstrual pain, and changes in the duration and amount of menstrual bleeding [41, 42]. These symptoms are accompanied by changes in patterns of ovarian and pituitary hormone secretion, such as an increase in follicular stage length and changes in follicular stimulating hormone (FSH) concentrations [8, 10, 42, 43]. These effects are apparent even when the studies controlled for health, lifestyle, or job environment (i.e., stress) [43]. 

Pregnancy outcomes are also affected by the working environment. Female shift workers have a higher risk of producing premature and/or low birth weight babies, spontaneous abortion and subfecundity [10, 44]. Flight attendants who worked while they were pregnant were twice as likely to have a spontaneous abortion when compared to flight attendants who did not work during their pregnancy [45]. Some studies on pregnancy outcomes in flight attendants indicate that this risk of miscarriage is moderate compared to the general population of women [45, 46]. In a mouse model of shift work, there was a significant reduction in the percent of animals that mated when they were housed in a 22 hours (11 h light  : 11 h dark) or 26 hours (13 h light  : 13 h dark) light-dark cycles for 2 or more weeks prior to mating [47]. Interestingly, if pregnant animals were taken from a 24 hours light:dark cycle and moved to a 22 or 26 hours light:dark cycle there was very little effect on the pregnancy outcomes. Entrainment to the light:dark cycle in women and rodents may be essential for successful copulation and conception, however, once pregnancy is achieved, female hormonal secretion patterns may be less sensitive to environmental changes [47]. 

Shift work and jet lag may exert their effects on health and physiology by reducing the total amount of sleep for an individual. Women working the night shift or experiencing transmeridian travel report a decrease in the amount of sleep and an increase in fatigue and insomnia [13, 41]. This reduction in sleep duration is not trivial as it has an effect on hormone secretion patterns. For example women with less than 8 hours of sleep secrete 20% less FSH compared to women with longer sleep durations [48]. Total or partial sleep deprivation increases LH amplitude, estradiol and FSH concentrations in normal cycling women [49]. Increased estrogen is associated with an increased risk of breast cancer (discussed below). It is possible that the altered menstrual cycle physiology associated with circadian misalignment is due to a direct effect of sleep state on ovarian and pituitary hormone secretion. It remains to be eluciated if circadian disruption, while significant to emotional well being and other physiological aspects, is a critical mechanism underlying the reproductive dysfunctions [41].

## 5. Breast Cancer and Biological Rhythms

In the last decade there has been a strong link between shift work and incidence of breast cancer. As a number of recent reviews discuss this issue in depth, this topic will only be highlighted here [50, 51]. There is a substantial literature which links light exposure at night, shift work or transmeridian travel, and an increased risk of breast cancer [9, 51–54]. One report examining over 85,000 women enrolled in the Nurses' Health Study found that a woman's relative risk of getting breast cancer was amplified if she worked the night shift or had rotating shifts [52, 55]. Similar risk levels have been determined for female flight attendants [53]. Animal cancer models indicate that altered circadian function exacerbates cancer symptomology. In a series of elegant studies, Filipski et al. disrupted circadian rhythms in mice either through SCN ablation or jet-lag schedules (repeated advances of the light:dark cycle). Mice experiencing this desynchrony had significantly accelerated growth of inoculated tumor cells [56–58].

One hypothesis which addresses the mechanism underlying this risk of breast cancer in shift workers is the “light at night theory” [51]. This postulates that the increased exposure to light during evening working hours decreases melatonin secretion. Melatonin is a pineal hormone that is secreted during the dark phase of the light:dark cycle and is suppressed when an individual is exposed to light including artificial light. Melatonin concentration, diurnal pattern of melatonin secretion, and the relationship of this pineal hormone rhythm to other physiological rhythms are altered in shift workers compared to daytime employees [13]. When compared to day-shift workers, women working the second or third shift have altered melatonin rhythms as measured by the urinary melatonin breakdown product 6-hydroxymelatonin sulfate [2]. This hormone has a protective effect against cancer, and can inhibit the growth of metastatic cells. In in vitro studies melatonin can suppress the growth of malignant breast cancer cells (reviewed in [59]). 

Melatonin-rich blood (collected at night from healthy women) suppresses tumor growth in immunodeficient rats carrying a human breast cancer xenograft. When these animals were given the melatonin rich blood and a melatonin receptor inhibitor, or blood collected during the day, the tumor suppressive effects were eliminated [50]. 

There is a strong link between light at night, melatonin, and breast cancer risk However, shift work or sleep deprivation may not be the direct cause of cancer. Rather the exposure to light at night suppresses the oncostatic hormone melatonin and accelerates the development of cancer symptoms. Additionally, as mentioned above, sleep deprivation can alter gonadal and pituitary hormone secretion patterns which may influence tumor cell growth. It is clear that shift work, jet lag, and sleep disturbances put a woman at increased risk for acquiring this pathology and this will require additional research to determine the causal relationships.

## 6. Clock Genes and Reproduction

The link between circadian rhythms and reproductive function also functions at the molecular level. In the last decade a family of “clock” gene and protein transcription and translation feedback loops have been identified. These clock genes play a role in an individual's rhythmicity, entrainment and responsiveness to light [60–62]. In mammals, the proteins CLOCK and BMAL1 form heterodimers. This complex then activates the transcription of thee *Period *genes known as *Per1, Per2, and Per3*. This CLOCK/BMAL1 heterodimer also turns on the transcription of two *cryptochome *genes known as *Cry1 *and *Cry2*. The protein products of the *Per *and *Cry *genes heterodimerize, then act as repressors and turn off the transcription of *Clock *and *Bmal1. *A second protein, NPAS2 also forms heterodimers with BMAL and this protein complex also initiates *Per* and *Cry *transcription [63]. 

The initial identification of rhythmic expression of clock gene transcription and translation was within individual cells of the SCN. These molecular rhythms have also been found in peripheral organs including female reproductive tissues such as the ovary [64, 65], uterus [66–68], and oviducts [69] (Figure 1).

Daily rhythms in clock gene expression have been found in an additional component of the HPG system; the GnRH neurons themselves. In cell cultures of GnRH GT1-7 cells, mRNA of *Clock, Bmal1, Per1 *and* Per2, *and the protein BMAL1 have a diurnal pattern of expression [70, 71]. Transfecting these cells with an altered CLOCK protein (Clock^Δ19^) or the addition of the CRY protein to the culture alters the amplitude and frequency of GnRH pulsatility [70]. The exact role of the molecular clock within these neuroendocrine cells has not been determined. It is possible that this is a mechanism which alters cellular activity of GnRH neurons, or modifies their sensitivity to estradiol [72]. 

Further evidence that clock gene rhythmicity is critical for reproductive function is seen in knock out or transgenic animal models. Disruptions in known circadian clock genes disrupts reproductive processes in female rodents; several detailed reviews have been published recently [19, 73]. In knock-out mice missing either the *Per1* or *Per2* gene, estrous cycles are irregular or absent, and animals have decreased fertility. This reduced fertility is more pronounced in “middle aged” mice compared to young mice, suggesting that mutations in this gene accelerate ageing at least with respect to reproductive function [74]. *Bmal1* knock-out female mice are able to mate but are not able to produce young [75]. Mice expressing a homozygous genotype of a mutated CLOCK protein (Clock^Δ19/ Δ19^) have a dampened LH surge, disrupted and irregular estrous cycles and difficulties with pregnancy [76]. Not all clock genes have an equal effect on reproduction as mice lacking*, Per3, Cry1, or Cry2 *(or combinations of these genes) are able to breed and reproduce [19]. 

Fertility and reproductive cyclicity may depend upon the precise phase relationship between the “master” clock contained in the SCN and the clocks contained within reproductive tissues and GnRH neurons. Clock gene rhythms within the SCN and peripheral tissues have a phase relationship. The peak expression of *Bmal1* in the rat ovary is about 4 hours delayed relative to the acrophase of *Bmal1* mRNA in the SCN. Similarly, the *Per2* rhythm in the ovary peaks 4 hours after lights off, whereas it peaks 6 hours earlier in the SCN [65]. If a female experiences a shift in the light:dark cycle, it is unknown how long it takes the circadian clock genes in the SCN, ovary and uterus to resynchronize to one another. It is known that in mice experiencing a phase advance, *Per1* mRNA rhythms in the SCN rapidly readjust to the new light:dark cycle but the peripheral organs (liver, lung, muscle) take nearly 6 times as long as the SCN to recover [77]. Rhythms in clock gene expression thus adjust to jet lag at different rates relative to one another, and relative to the peripheral organs. It is this mismatch of rhythms within the body that may underlie the reproductive deficits experienced by women experiencing disrupted biological rhythms [77, 78]. 

The relationship between clock genes and female health has not yet been examined closely in women; however, several studies have correlated circadian clock gene polymorphisms with reproductive disorders. The expression of three different polymorphisms of the NPAS2 gene was examined in control (*n* = 476) and breast cancer cases (*n* = 431). This gene is part of the transcription-translation loop of clock genes. A significant association was found between breast cancer risk and one of the heterozygous gene polymorphisms (compared to homozygous genotype) [63]. This same research group also found a 1.7 fold increased risk of breast cancer in women with a heterozygous genotype for a *Per3 *length polymorphism compared to women with a homozygous genotype [79]. In contrast no link was found between endometriosis, shift work, and the expression of a polymorphism of the *Clock* gene (hT3111C) in humans. This gene is correlated with mood disorders and in *Clock* mutant mice estrous cyclicity is impaired [76, 80]. Despite these data, the authors did find that women working the night shift had a nearly doubled increase in risk of endometriosis and this was further increased if women had altered sleep rhythms on their days off. Circadian gene markers may provide a valuable tool for identifying individuals in shift work environments that may be particularly susceptible to developing diseases. These markers may also help identify those individuals that may be better able to adapt to or accommodate a changing work schedule.

## 7. Interaction between Circadian Timing Mechanisms and Ovarian Hormones

A reciprocal interaction exists between the circadian timing mechanisms and gonadal hormones. As described above, the timing of estrus-related events including hormone secretion is regulated in part by the SCN and circadian system. Ovarian hormones in turn influence the behavioral and molecular circadian rhythms [81, 82]. On the day of sexual receptivity (estrus), female rats, hamsters, and degus (*Octodon degus, *an hystricomorph rodent) advance the onset of their daily activity rhythms [83–85]. Ovariectomized female hamsters and rats have given a capsule containing estrogen similarly advance the onset of their activity rhythms and have a shorter free running period when compared to control animals [86, 87]. 

Ovarian hormones appear to have a similar effect in women as diurnal and circadian rhythms including sleep-wake cycles and endocrine rhythms (cortisol, melatonin) change between the follicular and luteal phases of the reproductive cycle [88–90]. There are relatively few studies that have examined these rhythms in a controlled environment but one generalization is that ovarian hormones modify the amplitude but not the phase of various physiological rhythms. For example, humans have a daily rhythm in the fluctuation of body temperature, the nadir occurs after lights-off, and the temperature remains relatively low until the time of lights-on. In females, this general pattern remains consistent across the menstrual cycle but the amplitude of the rhythm is reduced during the luteal compared to follicular phase [91]. Similarly, when women were studied in an ultrashort sleep-wake protocol (which separates the endogenous rhythms from the influence of the environmental cues) the daily rhythm in cortisol was blunted during the luteal compared to follicular phase [90]. 

Ovarian hormones also influence the expression of circadian clock genes both within and outside of the SCN. Importantly, the effects of estrogen on the rhythm of clock gene expression are both tissue and gene specific. In ovariectomized female rats, chronic estrogen treatment significantly phase advances the acrophase of *Per2* mRNA expression, but not that of *Per1,* in the SCN [67]. An injection with estrogen significantly decreases the amount of *Cry2* mRNA within the SCN, but does not change the amount of *Cry1* mRNA [92]. In the uterus, estradiol treatment results in bimodal *Per1* and *Per2* expression whereas control animals had a single peak and estrogen shortens the period of *Per2 *expression [67, 93]. On the day of proestrus (high estradiol), *Bmal1* mRNA levels are increased relative to diestrus [65]. Data on clock gene expression in reproductive tissue of women is limited; breast and endometrial cancer lines and tissue from breast cancer patients express clock genes and their protein products [94, 95]. Additionally, *Per2* expression inhibits the expression of estrogen receptors in breast cancer cell lines [94]. Lastly, it is possible for estrogen to have a direct effect on the circadian timing system as estrogen receptors have been detected in the human SCN [96]. 

In rodent studies, changing concentrations of estradiol, either though the endogenous estrous cycle, or though disrupted ovarian function can phase shift or desynchronize circadian genes in both a tissue specific and clock gene specific manner [67]. This perturbation in the steroid hormone signal can lead to a change in the expression of circadian clock genes both within the SCN and in peripheral tissues including female reproductive organs. It will be important to determine if these factors also play a role in human reproductive health and disease.

## 8. Conclusions

The general mechanisms by which the SCN and circadian system regulate the physiological rhythms are still being elucidated. The investigation of specific central (SCN) or peripheral oscillators that regulate rhythms in the well-described hypothalamic-pituitary-gonadal system will provide a more general understanding of how the circadian clock mechanism regulates rhythmic outputs. Future work will also clarify the relationship between the circadian timing system and the contribution of other factors that impact women's reproductive health. Life or work stressors, sleep deprivation and fatigue, smoking habits, age, weight, and environmental conditions such as exposure to solvents all impact female reproductive function [97]. 

The circadian timing system and SCN regulate the onset of the preovulatory LH surge, ovulation, and mating behavior in rodents. Rhythmic clock gene expression within the SCN and peripheral reproductive tissues in females, and the relationship of these rhythms to one another, may be critical for successful reproduction. I hypothesize those disruptions in the endogenous circadian timing mechanism underlie reproductive deficits. In animal models, disruptions in these rhythms, as seen in transgenic and knockout mice, SCN lesioned animals, or individuals experiencing changes in the light:dark cycle, lead to changes in estrous cyclicity and altered patterns of hormonal secretion. The desynchrony of gene expression within a tissue and between central and peripheral tissues may also impact upon an individual's ability to establish phase relationships to environmental cues. In women, perturbations in daily rhythms, as occurs in shift work, jet lag, and sleep deprivation is associated with an increased menstrual cycle irregularity, increased risk of miscarriage, difficulty in conceiving, and a higher risk of breast cancer. Females' health and physiology may be particularly vulnerable to circadian disruption as the resulting changes in steroid hormones secretion patterns can further alter clock gene rhythms. Further investigations are needed to examine how reproductive cycles are regulated in women, the impact of disturbed biological rhythms on reproductive physiology, and how to reduce the health risks associated with altered rhythms.
